# ﻿*Sinocrassulaholotricha* (Crassulaceae), a new species from Sichuan, China

**DOI:** 10.3897/phytokeys.251.134633

**Published:** 2025-01-29

**Authors:** Zhi-Bai Xu, Jing Zhao, Rong-Juan Li, Hong Jiang, Jia-Guan Wang, Chao Chen

**Affiliations:** 1 School of South Asian and Southeast Asian Languages and Cultures, Yunnan Minzu University, Kunming, Yunnan, 650504, China; 2 School of Ecology and Environmental Science, Yunnan University, Kunming, 650504, Yunnan, China; 3 Chengdu Institute of Biology, Chinese Academy of Science, Chengdu 610041, China; 4 Key Laboratory of Tropical Forest Ecology, Xishuangbanna Tropic Botanical Garden, Chinese Academy of Sciences, Mengla 666303, Yunnan, China; 5 Yuanjiang Savanna Ecosystem Research Station, Xishuangbanna Tropical Botanical Garden, Chinese Academy of Sciences, Yuanjiang 653300, Yunnan, China

**Keywords:** Crassulaceae, Phylogeny, Rosette, Sempervivoideae

## Abstract

A new species of crassulacean acid metabolism, *Sinocrassulaholotricha*, is described based on specimen collected from Sichuan Province, Southwest China. The new species can be distinguished from its morphologically and phylogenetically related species by the hairy plants and lack of rosette. In the present study, a molecular phylogeny, taxonomic description, distribution information, and photographs of this new species are presented.

## ﻿Introduction

*Sinocrassula* A.Berger belongs to the Crassulaceae J. St.-Hil., the largest family in Saxifragales with 35 genera and ca. 1410 species (Eggli 2003; Thiede and Eggli 2007; Messerschmid et al. 2020). The family Crassulaceae contain three subfamilies, Crassuloideae Burnett, Kalanchoideae A.Berger and Sempervivoideae Arn. (Thiede and Eggli 2007). *Sinocrassula* is monophyletic and sister to *Kungia* K.T.Fu, then these two genera are together sister to the clade comprised of *Meterostachys* Nakai, *Orostachys* Fisch., and *Hylotelephium* H.Ohba in Sempervivoideae (Gontcharova et al. 2006; Messerschmid et al. 2020; Liu et al. 2023). *Sinocrassula* is a small genus consisting of ca. 13 species, mainly distributed in South and East Asia, with few species extending to Southeast Asia (Fu and Ohba 2001; Wang et al. 2012, 2022; Averyanov et al. 2014). Some species of *Sinocrassula* have been used as traditional Chinese medicines, such as *S.indica* (Decne.) A. Berger, which was often used to treat rheumatic arthritis, stomach ache, and fracture (Zhao et al. 2004; Xie and Yoshikawa 2012).

During a field trip in Sichuan, China, some special materials of *Sinocrassula* from two populations caught our attention. These materials were covered with hairs on the whole plants but lack a rosette, which is obviously different from the currently documented species in *Sinocrassula*. We executed the morphological and molecular study and confirmed that these materials represented an undescribed species. We describe and illustrate it here as *Sinocrassulaholotricha* J. Guan Wang, Jing Zhao & Chao Chen.

## ﻿Materials and methods

Plants were grown in the greenhouse of Yunnan University. Their morphologies were observed, and photographs are taken using Camera (Nikon, Japan) and SMZ1270 stereo microscope (Nikon, Japan) from living plants. Morphological characteristics were measured using ImageJ (https://imagej.nih.gov/ij/).

Two samples, representing two populations of the new species, were used for the phylogenetic analysis (Table 1). We used 41 samples, including 27 genera from the Crassulaceae and 14 samples representing six species of *Sinocrassula*. Subfam. Kalanchoideae were selected as the outgroup based on the previous phylogenetic studies (Messerschmid et al. 2020; Wang et al. 2022).

**Table 1.** List of taxa sampled with information related to taxonomy, GenBank accession numbers, references, and voucher information. Herbarium acronyms follow Index Herbariorum (Thiers 2024).

Species	Locations	Vouchers	*mat*K	*psb*A-*trn*H	*rbc*L	*trn*L-*trn*F	ITS	Reference
*Sinocrassulaambigua*	Yunnan, China	Chen et al. YUS12973 (YUKU)	PQ629047	PQ629054	PQ629039	PQ629032	PQ611189	This study
*S.ambigua*	Yunnan, China	Chen et al. YUS6698 (YUKU)	PQ629048	PQ629059	PQ629040	PQ629035	PQ611190	This study
*S.ambigua*	Yunnan, China	Chen et al. YUS12672 (YUKU)	PQ629046	PQ629055	PQ629038	PQ629030	PQ611188	This study
*S.densirosulata*	Sichuan, China	Chang XC19075 (SZ)	MW206800	MW206800	MW206800	MW206800	–	Chang et al. 2021
*S.holotricha*	Sichuan, China	Zhao et al. YUS13475 (YUKU)	PQ629050	PQ629056	PQ629042	PQ629034	PQ611192	This study
*S.holotricha*	Sichuan, China	Zhao et al. YUS12867 (YUKU)	PQ629051	PQ629057	PQ629043	PQ629031	PQ611193	This study
*S.indica*	Yunnan, China	zjq20160061	MN794334	MN794334	MN794334	MN794334	–	Zhao et al. 2020
*S.jiaozishanensis*	Yunnan, China	Chen et al. JZS001 (YUKU)	MZ343261	MZ343262	MZ343263	MZ343264	MZ343260	Wang et al. 2022
*S.jiaozishanensis*	Yunnan, China	Chen et al. JZS002 (YUKU)	MZ343266	MZ343267	MZ343268	MZ343269	MZ343265	Wang et al. 2022
*S.jiaozishanensis*	Yunnan, China	Chen et al. YUS05900 (YUKU)	PQ629052	PQ629058	PQ629044	PQ629036	PQ611194	This study
*S.yunnanensis*	Yunnan, China	Chen et al. YUS13776 (YUKU)	PQ629049	PQ629061	PQ629041	PQ629033	PQ611191	This study
*S.yunnanensis*	Yunnan, China	Chen s.n. (HIB, Cult.)	KC988295	–	–	–	KC988288	Chen et al. 2014
*S.yunnanensis*	Yunnan, China	Mayuzumi C00115 (TI)	–	–	–	AB480669	AB088582	Mayuzumi and Ohba 2004
*S.yunnanensis*	Yunnan, China	Chen et al. YUS6697 (YUKU)	PQ629053	PQ629060	PQ629045	PQ629037	PQ611195	This study
*Kungiaaliciae*	China	Mayuzumi CH00061 (TI)	–	–	–	AB480632	AB480591	Mayuzumi and Ohba 2009
*Adromischusfallax*	Saudi Arabia	Bruyns 2997 (BOL)	MH503364	LN878728	–	LN878814	MH503497	Bruyns et al. 2019
*Aeoniumdecorum*	Gomera, Spain	Mort 1435 (WS)	AY082165	AY082197	–	AY082239	AY082130	Mort et al. 2002
*Aichrysonpachycaulon*	Spain	Mort 1404 (WS)	AY082157	AY082182	–	AY082223	AY082105	Mort et al. 2002
*Cotyledonbarbeyi*	Kenya	Bruyns 12754 (BOL)	MH503487	–	–	MH503217	MH503623	Bruyns et al. 2019
*Dudleyapulverulenta*	Mexico	Oceguera s.n. (XAL)	–	–	–	–	EF632171	Messerschmid et al. 2020
*Echeveriaamoena*	Mexico	Carrillo-Reyes & Nicolalde 4233 (IEB, XAL)	–	–	–	–	EF632172	Messerschmid et al. 2020
*Graptopetalumamethystinum*	Mexico	Acevedo 1734 (XAL and NYBG)	–	–	–	–	AY545690	Messerschmid et al. 2020
*Hylotelephiumtatarinowii*	China	Zhang 100717-08 (PEY)	–	KF113734	–	KF113787	KF113681	Zhang et al. 2014
*Kalanchoegracilipes*	Madagascar	Bruyns 6232 (BOL and MO)	MH503489	–	–	MH503219	MH503625	Bruyns et al. 2019
*K.pinnata*	Florida, USA	Davis 1290 (FLAS)	GU135118	GU135449	GU135277	–	–	Messerschmid et al. 2020
*Lenophyllumacutifolium*	New York Botanical Garden	Rose s.n. (NYBG)	–	–	–	–	AY545709	Messerschmid et al. 2020
*Meterostachyssikokianus*	Nagasaki, Japan	Mayuzumi et al. C00028 (TI)	–	–	–	AB480670	–	Mayuzumi and Ohba, unpublished
*Monanthesadenoscepes*	Tenerife, Spain	Santos s.n.	AY082264	AY082277	–	AY082291	AY082118	Mort et al. 2002
*Orostachysmalacophylla*	Primorsky, Russia	Mayuzumi CH00054B (TI)	–	–	–	AB480617	AB480580	Mayuzumi and Ohba, unpublished
*Pachyphytumfittkaui*	Mexico	HBG-49458	–	–	–	–	FJ753925	Messerschmid et al. 2020
Petrosedumamplexicaulesubsp.tenuifolium	Spain	MJG 024790	MT181567	–	–	–	MT336100	Messerschmid et al. 2020
*Phedimusaizoon*	Hebei, China	Zhang et al. 120613-03 (PEY)	–	KF113735	–	KF113788	KF113682	Zhang et al. 2014
*Prometheumchrysanthum*	Turkey	Stephenson 4R022	KX452252.1	–	–	–	HE999634	Messerschmid et al. 2020
*Pseudosedumlievenii*	Turkmenistan	Regel 1079 (US)	–	–	–	–	KJ569920	Zhang et al. 2014
*Rhodiolahumilis*	Xizang, China	Zhang et al. 110804-03-03 (PEY)	KP114838	KP114937	KP115042	KP115148	KP114742	Zhang et al. 2014
*Sedumalfredii*	China	Kokubugata 17191 (TNS)	–	–	–	LC229500	AB930260	Messerschmid et al. 2020
*Sempervivumtectorum*	Canada	CCDB-18313-F08	–	–	MG249291	–	MG237296	Kuzmina et al. 2017
*Thompsonellacolliculosa*	Mexico	Carrillo-Reyes & Pérez-Calix 2714 (IBUG, IEB, and GUADA)	–	–	–	–	EF632177	Messerschmid et al. 2020
*Tylecodonracemosus*	Namibia	Bruyns 9476b (BOL)	–	–	–	–	MH503627	Messerschmid et al. 2020
*Umbilicusschmidtii*	Cape Verde	Romeiras & Carine 3170 (LISC)	KP279381	KP279450	–	KP279339	–	Romeiras et al. 2015
*Villadiadiffusa*	Mexico	Nicolalde 1461 (XAL)	–	–	–	–	FJ753973	Messerschmid et al. 2020

The TIANGEN plant genomic DNA extraction kit (TIANGEN Biotech., Beijing, China) was used to extract total genomic DNA from silica-dried material, following the manufacturer’s protocols. We selected four plastid markers (*rbc*L, *mat*K, *psb*A-*trn*H, and *trn*L-*trn*F) and one nuclear gene (ITS) for amplification and sequencing. The primers and PCR conditions of Zhang et al. (2015) and Kress and Erickson (2007) were used. After amplification, the fragments were purified using TIANquick Mini Purification Kits (Tiangen Biotech, Beijing, China), and the purified PCR products were sequenced by Tsingke (Beijing, China).

The newly generated sequences were edited and assembled using Sequencher v.4.1.2 (Gene Codes Corporation, Ann Arbor, Michigan). Subsequently, all the sequences were aligned using MAFFT v.7 (Katoh and Standley 2013), with manual adjustments in BioEdit (Hall 1999). Maximum likelihood (ML) bootstrapping was conducted with 1000 rapid bootstrap (BS) replicates, followed by a search for the best-scoring tree in a single run using RAxML v.8 (Stamatakis et al. 2008). Bayesian inference (BI) was conducted using MrBayes v.3.1.2 (Huelsenbeck and Ronquist 2001), with two runs of four Markov chain Monte Carlo chains. Each run began with a random tree and sampled one tree every 1,000 generations for 2,000,000 generations. Both ML and BI analyses were conducted on the Cipres Science Gateway (Miller et al. 2010).

## ﻿Results and discussion

**Figure 1.**
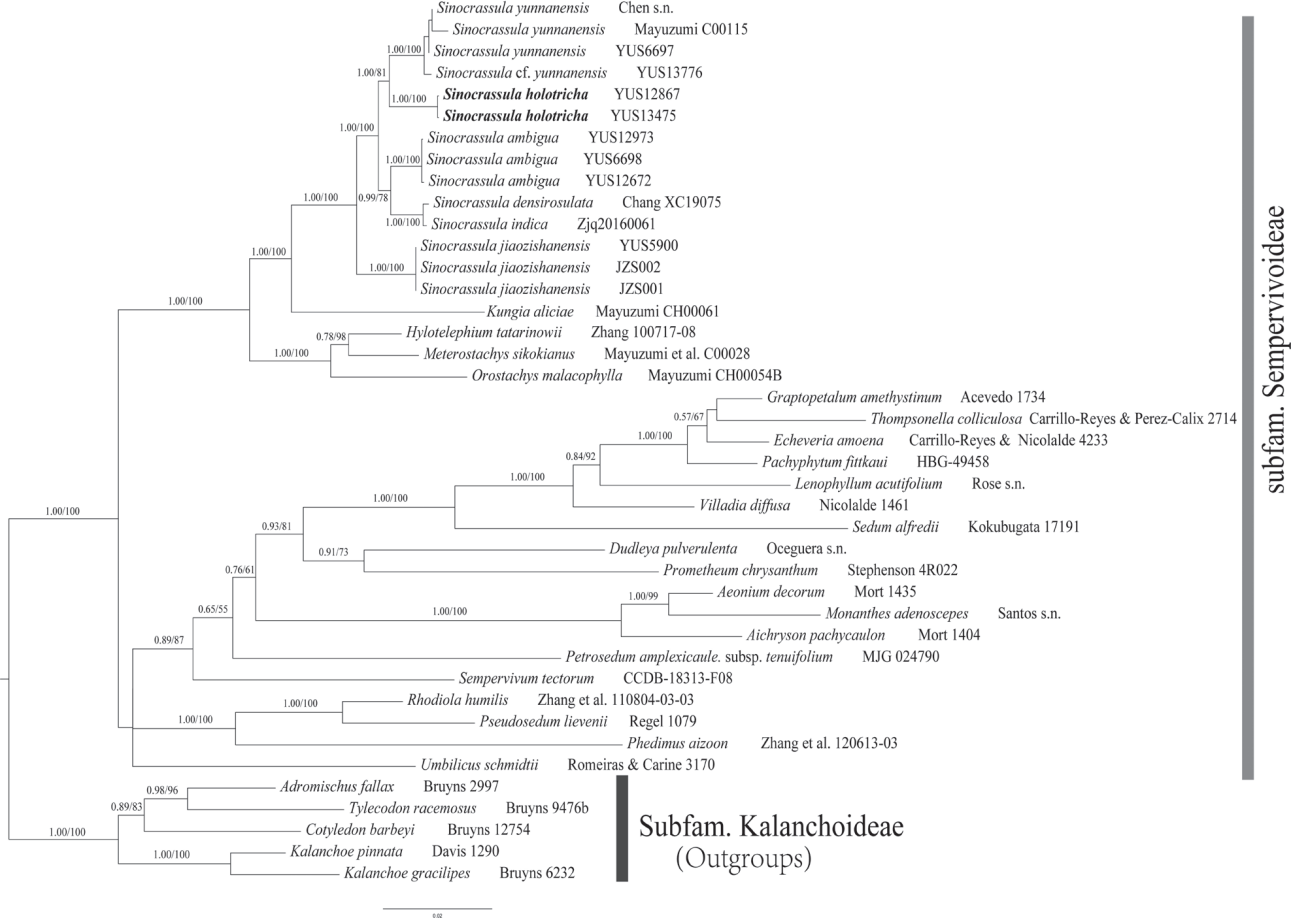
The maximum likelihood phylogeny of *Sinocrassulaholotricha* and its allies based on four plastid markers (*rbc*L, *mat*K, *psb*A-*trn*H, and *trn*L-*trn*F) and one nuclear gene (ITS). Support values of Maximum likelihood bootstrap support and Bayesian inference posterior probability are shown above the main branches.

In our phylogeny, six *Sinocrassula* species with 14 accessions were included in this study (Fig. 1, Table 1). *Sinocrassulajiaozishanensis* Chao Chen, J. Guan Wang & Z. R. He formed the first divergent clade in *Sinocrassula* with maximum support, and two samples of the new species was resolved as sister to *S.yunnanensis* (Fig. 1).

Morphologically, the new species is similar to *Sinocrassulayunnanensis* in having dense and short hairs on the whole plants. In *Sinocrassula*, except the new species, *S.yunnanensis* is another species covered with hairs on whole plants. The rest of the species in *Sinocrassula* are entirely glabrous or hairy on leaves or flowering stems only (Fu and Ohba 2001). However, in our phylogeny, *S.yunnanensis* is sister to *S.ambigua* but is a distant relative of the new species (Fig. 1). The rosette is a very important morphological character in *Sinocrassula* which has always been used to identify species (Fu and Ohba 2001; Thiede and Eggli 2007; Wang et al. 2022). Except for the new species, only two other species (*S.ambigua* and *S.jiaozishanensis*) lack the rosette in the current documented species (Table 2). However, phylogenetic analysis showed that these three species have a distant phylogenetic relationship from each other (Fig. 1). Potentially, the lack of a rosette could be the ancestral character in *Sinocrassula*. Based on the phylogeny and specific morphological characters, we describe the new species as *S.holotricha* herein.

**Table 2.** A morphological comparison among *Sinocrassulaambigua*, *S.holotricha*, *S.jiaozishanensis*, and *S.yunnanensis*.

Character	*S.ambigua*	*S.holotricha*	*S.jiaozishanensis*	*S.yunnanensis*
**Life cycle**	Perennial	Annual	Perennial	Annual or Biennial
**Basal leaves**	Not rosulate	Not rosulate	Not rosulate	Rosulate
**Plant surface**	Glabrous	With short pubescence	Glabrous	With short pubescence
**Leaves**	Beige to purplish red, glabrous	Green to purplish black, with short pubescence	Turquoise with red edge, glabrous	Green to purplish black, with short pubescence
**Bracts**	Linear-oblong	Obovate-lanceolate	Lanceolate to oblanceolate	Obovoid-lanceolate
**inflorescences length**	1.2–2.5 cm	5–10 cm	15–20 cm	5–10 cm
**Flowers color**	Reddish purple	Orangish-red	Reddish purple	Yellowish green
**Nectar scales**	Subquadrate	Ligulate	Oblong	Quadrate
**Nectar scales size**	0.5 × 0.5 mm	0.45–0.50 × 0.25–0.30 mm	0.3 × 0.6 mm	Unknown
**Phenology**	May. –Jul.	Jun. –Oct.	Mar. –June.	Sept. –Oct.

### ﻿Taxonomic treatment

#### ﻿ Sinocrassula holotricha

Taxon classificationPlantaeSaxifragalesCrassulaceae

J. Guan Wang, Jing Zhao & Chao Chen sp. nov.

5C766CB3-A624-5F52-82B3-C953A0917625

urn:lsid:ipni.org:names:77355915-1

Figs 2 , 3

##### Type.

China • Sichuan: Jiulong County, elev. ca. 2384 m, 28.832849°N, 101.612746°E, on the granite crevices, 7 July 2023, *Jing Zhao et al. YUS-13475* (holotype: YUKU!; isotypes: YUKU!).

##### Diagnosis.

Morphologically, *Sinocrassulaholotricha* is similar to *S.ambigua* and *S.jiaozishanensis*, but clearly differs from the latter two by its hairy plants (vs. glabrous), and similar to *S.yunnanensis* (Franch) A. Berger in having hairy plant, but differs from the latter in basal leaves opposite (vs. rosette), orangish-red (vs. yellowish green) petals, and ligulate (vs. quadrate) nectar scales (Table 2). The new species with the combined morphological characters of hairy plants and lack of basal rosette is obviously different from these known species.

##### Description.

**Figure 2.**
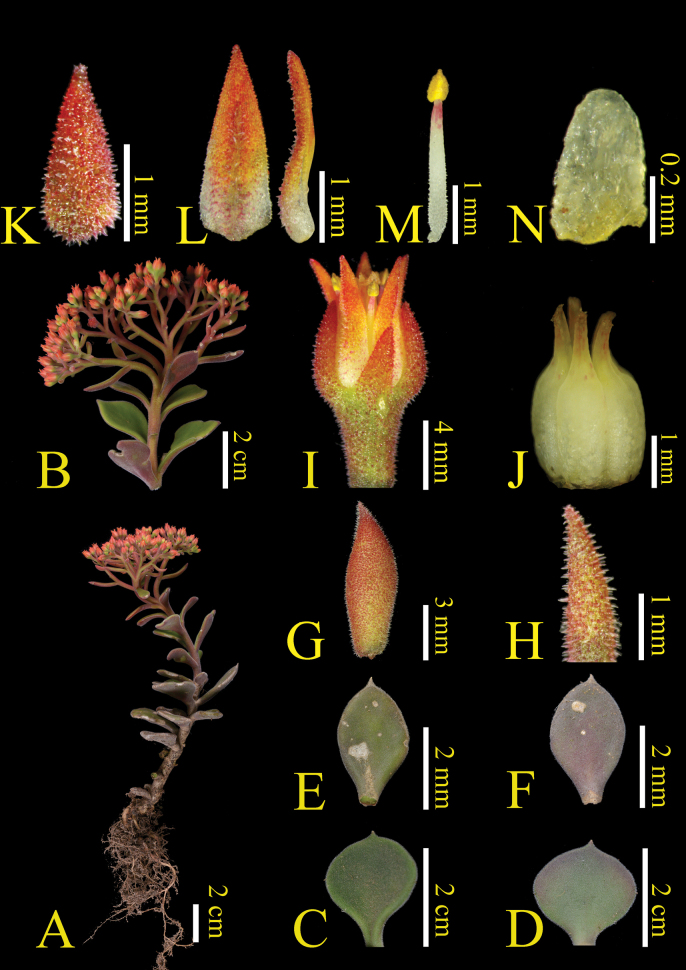
*Sinocrassulaholotricha***A** habit **B** inflorescence **C**, **D** basal leaves **E**, **F** stem leaves **G, H** bracts **I** flower **J** carpels **K** sepal **L** petals **M** stamen **N** nectar scale.

**Figure 3.**
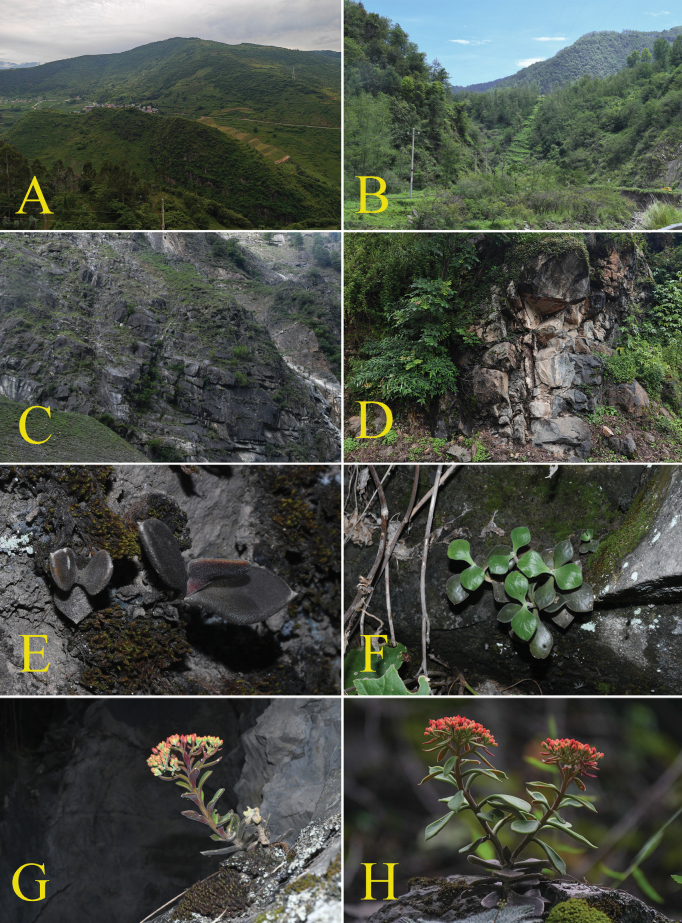
*Sinocrassulaholotricha***A–D** habitats **E, F** immature plants **G, H** flowering plants.

Plants terrestrial or lithophytic, perennial, 15–20 cm tall, hairy throughout (Figs 2A, 3G, H). Roots fibrous. Leaves without basal rosettes, basal leaves opposite, oblanceolate-rounded, 2.0–2.5 × 2.0–2.5 cm, stem leaves oblanceolate -oblong, 2.5–3.0 × 1.2–1.5 cm, apex cuspidate, (Figs 2A, 3G, H). Sterile stems short, 8.0–15.0 cm tall, simple, slightly thickened to the base, 5.0–7.0 mm in diam. (Fig. 2A). Flowering stems elongated, 5–10 cm, sparely leafy, hairy; stem leaves nearly opposite, ± orbicular at base, upward obovate, hairy (Fig. 2A, B). Inflorescences corymbiform, densely orange papillate, ca. 7.0 cm in diam. (Fig. 2A, B); bracts few, obovate-lanceolate, pubescent and glandular (Fig. 2G, H). Flowers small, ca. 4.5–5.0 × 3.5–4.0 mm in diam; pedicels purple, slightly longer than flowers (Fig. 2A, B, I). Sepals obovate-triangle, ca. 2.5–3.5 × 1.0–1.5 mm, minutely and densely orange papillate, apex obtuse, base rounded (Fig. 2K). Petals yellowish white, deeply orange upward, ovate-lanceolate, 3.5–4.2 × 1.0–1.5 mm, minutely papillate abaxially (Fig. 2L). Stamens slightly shorter than petals, ca. 3.0 mm (Fig. 2M). Nectar scales nearly ligulate, ca. 0.45–0.50 × 0.25–0.30 mm (Fig. 2N). Carpels 5, lanceolate, 3.0–3.3 mm (Fig. 2J). Styles short, ca. 0.5–0.7 mm (Fig. 2J). Flower Jun. –Oct.

##### Distribution and habitat.

*Sinocrassulaholotricha* is known only from the west and south west Sichuan in China. Two populations were found on granite crevices, dry stony, or gravelly slopes at elevations of ca. 1500–2400 m (Fig. 3).

##### Additional specimens examined.

**China** • **Sichuan**: Yuexi County, elev. ca. 1522 m, 28.704771°N, 102.596450°E, on the granite crevices, 5 July 2023, *Jing Zhao et al. YUS-12867* (YUKU!).

##### Etymology.

The specific epithet *holotricha* is derived from the Latin *holo*, meaning whole, and “trichome,” meaning hair, in reference to the plants covered by pubescence throughout.

## Supplementary Material

XML Treatment for Sinocrassula holotricha

## ﻿Additional information

### Author ORCIDs

Zhi-Bai Xu https://orcid.org/0009-0006-3565-0777

Jing Zhao https://orcid.org/0000-0001-7871-2209

Rong-Juan Li https://orcid.org/0009-0008-2667-398X

Hong Jiang https://orcid.org/0000-0002-3509-3708

Jia-Guan Wang https://orcid.org/0000-0003-0264-9180

AuthorName https://orcid.org/0000-0001-9533-7340

### Data availability

All of the data that support the findings of this study are available in the main text.
